# NeisseriaBase: a specialised *Neisseria* genomic resource and analysis platform

**DOI:** 10.7717/peerj.1698

**Published:** 2016-03-17

**Authors:** Wenning Zheng, Naresh V.R. Mutha, Hamed Heydari, Avirup Dutta, Cheuk Chuen Siow, Nicholas S. Jakubovics, Wei Yee Wee, Shi Yang Tan, Mia Yang Ang, Guat Jah Wong, Siew Woh Choo

**Affiliations:** 1 Department of Oral Biology and Biomedical Sciences, Faculty of Dentistry, University of Malaya, Kuala Lumpur, Malaysia; 2 Genome Informatics Research Laboratory, HIR Building, University of Malaya, Kuala Lumpur, Malaysia; 3 Computer Science and Engineering Department, University of NE-Lincoln, Lincoln NE, United States of America; 4 Centre for Oral Health Research, School of Dental Sciences, Newcastle University, Newcastle upon Tyne, United Kingdom; 5 Genome Solutions Sdn Bhd, Suite 8, Innovation Incubator UM, Level 5, Research Management & Innovation Complex, University of Malaya, Kuala Lumpur, Malaysia

**Keywords:** NeisseriaBase, *Neisseria*, Genomic resources, Comparative analysis, Pairwise Genome Comparison tool, Pathogenomics Profiling tool

## Abstract

**Background.** The gram-negative *Neisseria* is associated with two of the most potent human epidemic diseases: meningococcal meningitis and gonorrhoea. In both cases, disease is caused by bacteria colonizing human mucosal membrane surfaces. Overall, the genus shows great diversity and genetic variation mainly due to its ability to acquire and incorporate genetic material from a diverse range of sources through horizontal gene transfer. Although a number of databases exist for the *Neisseria* genomes, they are mostly focused on the pathogenic species. In this present study we present the freely available NeisseriaBase, a database dedicated to the genus *Neisseria* encompassing the complete and draft genomes of 15 pathogenic and commensal *Neisseria* species.

**Methods.** The genomic data were retrieved from National Center for Biotechnology Information (NCBI) and annotated using the RAST server which were then stored into the MySQL database. The protein-coding genes were further analyzed to obtain information such as calculation of GC content (%), predicted hydrophobicity and molecular weight (Da) using in-house Perl scripts. The web application was developed following the secure four-tier web application architecture: (1) client workstation, (2) web server, (3) application server, and (4) database server. The web interface was constructed using PHP, JavaScript, jQuery, AJAX and CSS, utilizing the model-view-controller (MVC) framework. The in-house developed bioinformatics tools implemented in NeisseraBase were developed using Python, Perl, BioPerl and R languages.

**Results.** Currently, NeisseriaBase houses 603,500 Coding Sequences (CDSs), 16,071 RNAs and 13,119 tRNA genes from 227 *Neisseria* genomes. The database is equipped with interactive web interfaces. Incorporation of the JBrowse genome browser in the database enables fast and smooth browsing of *Neisseria* genomes. NeisseriaBase includes the standard BLAST program to facilitate homology searching, and for Virulence Factor Database (VFDB) specific homology searches, the VFDB BLAST is also incorporated into the database. In addition, NeisseriaBase is equipped with in-house designed tools such as the Pairwise Genome Comparison tool (PGC) for comparative genomic analysis and the Pathogenomics Profiling Tool (PathoProT) for the comparative pathogenomics analysis of *Neisseria* strains.

**Discussion.** This user-friendly database not only provides access to a host of genomic resources on *Neisseria* but also enables high-quality comparative genome analysis, which is crucial for the expanding scientific community interested in *Neisseria* research. This database is freely available at http://neisseria.um.edu.my.

## Introduction

The genus *Neisseria* is a member of the family of *Neisseriaceae* and shares the family along with three other genera: *Moraxella, Kingella, and Acinetobacter*. *Neisseria* are Gram-negative, aerobic diplococci and are fastidious organisms that grow at 37 °C. Unlike the Enterobacteriaceae and many other Gram-negative bacteria, *Neisseria* are oxidase-positive (Gordon & McLeod, 1928). The two most significant *Neisseria* pathogens which often cause disease in humans are *N. gonorrhoeae*, and *N. meningitidis.* Although they are genetically very similar, they have specialized to adapt to very different niches. *N. meningitidis* colonizes the nasopharynx of around 10–35% of adults (Read, 2014), and causes serious diseases such as septicaemia or meningitis. On the other hand, the gonococcus primarily colonizes the mucous membranes of the urethra in males and the endocervix and urethra in females, and is responsible for around 300,000 cases of sexually transmitted diseases per year in the US (Centers for Disease Control and Prevention, 2013).

The majority of human-associated *Neisseria* species are non-pathogenic and are part of the normal flora of the upper respiratory tract. They form the commensal flora of mucosal membranes of humans where they colonize the host without causing disease. However, commensal *Neisseria* species tend to be opportunistic pathogens causing infections and invasive diseases, especially in individuals with underlying medical conditions and/or immune suppression or deficiency. Commensal *Neisseria* species have been reported to be involved in bacteremia, endocarditis, meningitis, and septic arthritis. There are reports of *N. subflava*, *N. flava*, *N. perflava*, *N. mucosa*, and *N. sicca* being isolated from infectious processes, including endocarditis, bacteraemia, meningitis, pneumonia, empyema, pericarditis, peritonitis, septic arthritis, and liver abscess (Roberts et al., 2003; Chung, Teng & Hsueh, 2007; Janda & Gaydos, 2007). Three patients on continuous ambulatory peritoneal dialysis in Europe have been reported with *N. sicca/subflava* peritonitis (Vermeij et al., 1999; Konner et al., 2001). *N. cinerea* a common isolate from the upper respiratory tract, has also been isolated from other sites including the cervix, rectum, conjunctivae, blood and cerebrospinal fluid (CSF) (Janda & Gaydos, 2007) and has also been associated with rare cases of peritonitis (Taegtmeyer et al., 2006), tonsillitis, lymphadenitis, proctitis, and pulmonary cavitation (Kamar et al., 2007). *N. elongata* subspecies have been isolated from infectious processes, including endocarditis, septicaemia, and osteomyelitis (Haddow et al., 2003; Janda & Gaydos, 2007; Hsiao et al., 2008). There are reports of a case where *N. weaveri* was isolated from a lower respiratory tract infection (Panagea et al., 2002). There have also been reports of bacteramemic pneumonia and septicemia in immunocompromised individuals (Schifman & Ryan, 1983) caused by *N. lactamica*. Interestingly, some of the virulence factors of the pathogenic *Neisseria* such as iron scavenging systems and immunoreactive cell surface proteins are also present in commensal species, presumably because they promote the colonization of mucosal surfaces (Rotman & Seifert, 2014).

*Neisseria* are unique for their full competency to uptake DNA throughout their life cycle. Numerous studies have revealed that these transformations have caused recombinational exchanges between *Neisseria* species which has resulted in differences of nucleotide identity as high as 25% (Spratt et al., 1989; Bowler et al., 1994; Feil, Carpenter & Spratt, 1995; Zhou, Bowler & Spratt, 1997; Rotman & Seifert, 2014). There is even evidence that *N. gonorrhoeae* has acquired genetic information from human cells (Anderson & Seifert, 2011). These observations make it important to have a clearer understanding of the dynamic nature of the genus *Neisseria* at the genomic level.

To enhance *Neisseria* research, a specialized database system for *Neisseria* is critical for the storage of the genome sequences, annotations and for analytical purposes, particularly in the field of comparative genomics. Comparative genome analysis of *Neisseria* will have a deep impact on better understanding of the biology, diversity, evolution, and virulence of *Neisseria*. Although several *Neisseria* resource databases exist (Jolley & Maiden, 2010; Katz et al., 2011), their focus is primarily on the pathogenic clades of *Neisseria*. In this present study we describe NeisseriaBase, a freely available database with customised bioinformatics tools, dedicated to the genus *Neisseria*, encompassing the entire spectrum of *Neisseria* species (both pathogenic and commensal species). This database has been designed to provide resources for whole-genome annotations, facilitate comparative genomic analysis between the different *Neisseria* strains and computational predictions specifically designed to support the expanding *Neisseria* research community.

## Materials and Methods

### Data source

The genomic data of 227 *Neisseria* strains encompassing 15 species (including both complete and incomplete genomes) were retrieved from National Center for Biotechnology Information (NCBI) GenBank for further annotation and analyses (Table 1).

**Table 1:** List of available *Neisseria* strains/genome sequences in NeiseriaBase.

Sl. no.	Species	Number of genomes	
		Draft	Complete
1	*N. bacilliformis*	1	0
2	*N. cinerea*	1	0
3	*N. elongata*	1	0
4	*N. flavescens*	2	0
5	*N. gonorrhoeae*	17	3
6	*N. lactamica*	2	1
7	*N. macacae*	1	0
8	*N. meningitidis*	171	14
9	*N. mucosa*	2	0
10	*N. polysaccharea*	2	0
11	*N. shayeganii*	1	0
12	*N. sicca*	4	0
13	*N. subflava*	1	0
14	*N. wadsworthii*	1	0
15	*N. weaveri*	2	0

### Annotating and analysing the genome sequences

To ensure the uniformity in the annotations of the genomes retrieved from NCBI GenBank, all 227 genome sequences (both complete and incomplete) were annotated by using a fully annotated web-based engine, the Rapid Annotation using Subsystem Technology (RAST) pipeline (Aziz et al., 2008). The RAST pipeline is capable of predicting genes by identifying elements on the genome such as protein-encoding genes and RNA genes (Aziz et al., 2008). Functional protein assignments were provided by RAST, where proteins were predicted based on their relatedness within the subsystems in FIGfams database. The latest PSORTb version 3.0 was used to systematically determine the subcellular localizations for each putative protein (Yu et al., 2010). Further analyses such as calculation of GC content (%), hydrophobicity and molecular weight calculation of the predicted genes were performed using a number of in-house scripts developed through BioPerl. All the results of the analysis were stored in the NeisseriaBase.

### Database system

A relational database was implemented by using MySQL version 14.12 (http://www.mysql.com). All the predicted CDSs and RNAs of all the *Neisseria* strains and all their related information such as the gene functions, GC content hydrophobicity and molecular weight were organised into a database schema and stored into the MySQL database.

### NeisseriaBase implementation

To provide a user-friendly interface to enable the easy accessing of the genomic information of interest, the system was designed following secure four-tier web application architecture: (1) client workstation, (2) web server, (3) application server, and (4) database server. All requests from web clients and the interactions with the back-end servers to implement the requests are handled by a dedicated Apache web server. The client workstations can interact with the web server where the website is hosted using modern web browsers. The website was constructed using PHP and CSS, utilizing the model-view-controller (MVC) framework to separate the application data, presentation and logic into three distinct modules.

A feature-rich JavaScript library jQuery was used for Client-side scripting enhancing the user interaction with the web pages. It uses Asynchronous JavaScript and XML (AJAX) communication libraries for transmitting data between the client workstations and server-side programs in the background. A Linux server (CentOS 5.8) using in-house Perl, Python and R scripts, creating complex pipelines of inputs and outputs for the necessary programs, perform the server-side operations. MySQL was used to construct relational database to store annotated sequence data.

### A Pathogenomics Profiling Tool (PathoProT) for distinguishing virulence factors

All the experimentally verified virulence factors from different pathogenic organisms, curated in the Virulence Factor Database (VFDB) (VFDB Version 2012 containing a total of 19,775 proteins) (Chen et al., 2005; Yang et al., 2008; Chen et al., 2012) were retrieved for the PathoProT and treated as the “known virulence factors.” The orthologs of these known virulence factors or the “predicted virulence genes” are identified in the *Neisseria* genomes present in the NeisseriaBase by using the well-established BLAST (Stand-alone) tool of NCBI (Altschul et al., 1990; McGinnis & Madden, 2004; Camacho et al., 2009; Tao, 2010) embedded in the PathoProT pipeline. The default parameters for the BLAST search for the prediction of the orthologs are set to 50% sequence identity and 50% sequence completeness; however, these parameter values can be altered by the user depending on their desired levels of stringency. PathoProT is supported by in-house Perl and R scripts pipeline in such a way that Perl is used for initial process management whereas R is responsible generating hierarchical clustering of the identified virulence genes and producing a heat map of multiple virulence gene profiles for data visualization. The working of the PathoProT is explained in a flow diagram (Fig. S1).

### PGC tool: a pairwise genome comparison and visualisation tool

The alignment algorithm utilized in PGC is based on the NUCmer (global alignment) package in MUMmer 3.0, which is primarily used for large-scale and rapid genome alignment (Kurtz et al., 2004). The genomic comparison results will be processed by our PGC pipeline before they are displayed via a visualization tool called Circos (Circular Genome Data Visualization) (Krzywinski et al., 2009) which presents the similarities and differences arising from the comparison of the genomes in a circular ideogram layout. The three main parameters for PGC tool are the minimum percent identity (%), the link threshold (LT) (bp) which removes the links according to user-defined value, and the merge threshold (MT) (bp) which allows merging of links based on user-defined value. By default, the thresholds of the PGC tool are set to be 95% minimum percent identity and 1,000 bp link threshold. However, users may change these parameter values depending on their requirements to get different comparative results. The PGC plot generated displays the variation of genomic structures between two genomes of interest which relate to different positions such as conservation, symmetry, and rearrangements. These high resolution PGC plot results of the genome comparison can be downloaded in svg format from the NeisseriaBase.

### Sequence similarity search for NeisseriaBase and VFDB

The sequence similarity search of the query sequences can be performed by using the NCBI BLAST software package (Altschul et al., 1990; Altschul et al., 1997; McGinnis & Madden, 2004), which has been incorporated into the NeisseriaBase. This software package incorporated into NeisseriaBase will allow the users to search their sequences against the databases of interest. NeisseriaBase provides two variations of sequence search applications: BLAST and VFDB BLAST. These BLAST searches use similar approaches, which involve comparison of the regions of homology between the nucleotide or amino acid sequences (Altschul et al., 1990; Altschul et al., 1997; McGinnis & Madden, 2004). The standard BLAST search allows users to search a query protein or nucleotide sequence against all *Neisseria* genomes or a single or multiple genomes or in the case of nucleotide search, against genomic sequences or protein coding sequences only present in the NeisseriaBase. On the other hand, the VFDB BLAST search allows users to search a query sequence against the Virulence Factor Database (VFDB) (Chen et al., 2005; Yang et al., 2008; Chen et al., 2012) which would be useful in identifying potential virulence genes based on their sequence homology (Baumberg et al., 1995; Maiden, 2008; Marri et al., 2010).

### Availability and requirements

NeisseriaBase is available online at http://neisseria.um.edu.my. All sequences and annotations described in this paper can be downloaded from that site. NeisseriaBase is best viewed by Mozilla Firefox^®^ 10.x or higher, Safari 5.1 or higher, Chrome 18 or higher and any other equivalent browser software. If the browser is older, the user may have trouble viewing some of our website features properly. This website is best viewed at a screen resolution of 1,024 × 768 pixels or higher.

## Results

### Database organization and features

**Figure 1:**
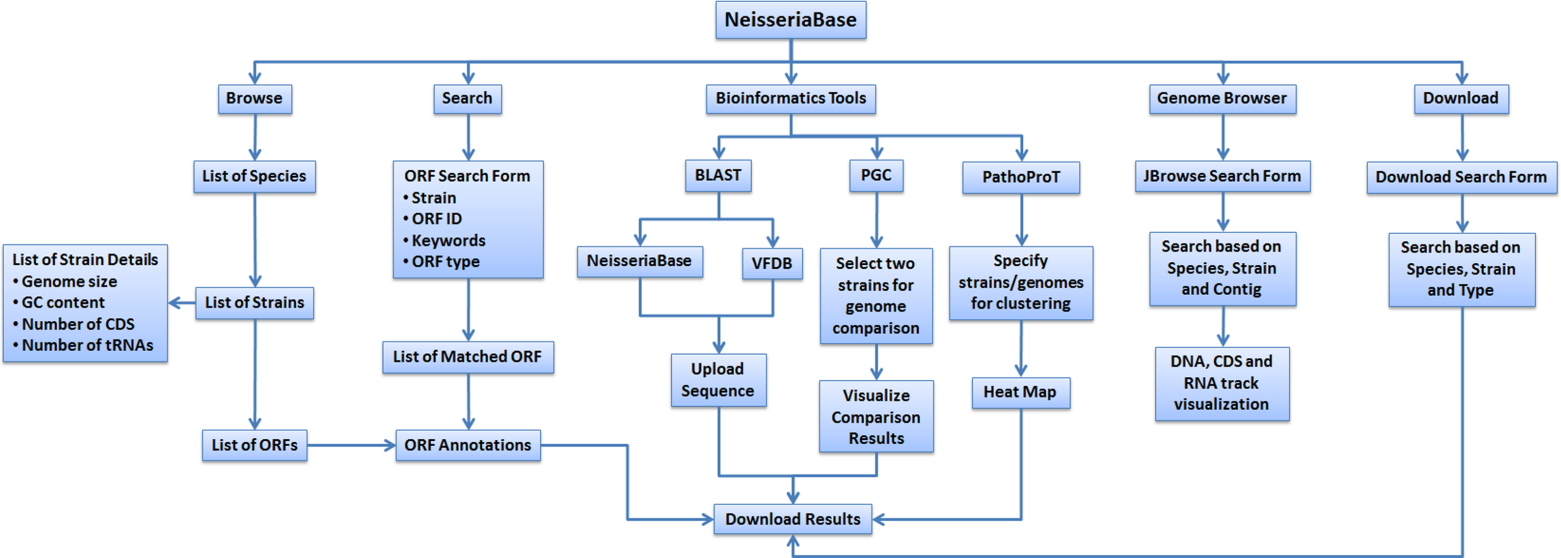
Flow diagram showing the overview of functionalities of NeisseriaBase.

The NeisseriaBase is a user-friendly online resource dedicated to the *Neisseria* genus, and is equipped with flexible bioinformatics applications and tools. An overview of the functionalities in NeisseriaBase is shown in Fig. 1. Detailed information for 227 *Neisseria* genomes is available in this database. Of the 227 sequenced genomes, 18 were whole genomes, whereas the rest of the strains were draft genomes (Table 1). The pathogenic species of *Neisseria*, *N. meningitidis* and *N. gonorrhoeae*, contributed a significant amount of genomic data: 185 strains were of *N. meningitidis* (14 whole genomes and 171 draft genomes) while 20 strains were grouped under *N. gonorrhoeae* (3 whole genomes and 17 draft genomes). Each strain was stored in accordance with their corresponding species.

**Figure 2:**
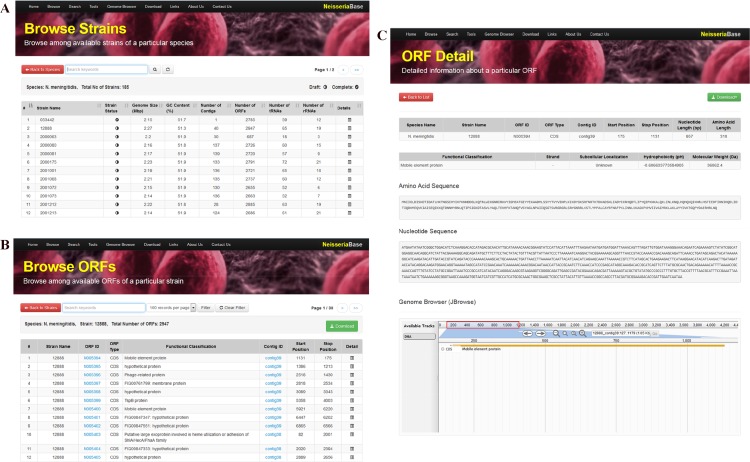
Overview of NeisseriaBase. (A) The database overview displaying the main list of all the strains of *Neisseria* along with the details (Status, Genome Size (Mbp), GC Content (%), Number of Contigs, Number of CDS, Number of tRNAs and Number of rRNAs) organized in columns; (B) List of all the CDSs and RNAs of a specific strain; (C) Detailed information of a CDS with visualization in JBrowse.

The list of species along with their total number and genome status are displayed in a tabular form. Each *Neisseria* strain has been attached with its related details such as the genome size, number of coding sequences, number of tRNAs and rRNAs, genome identity and coverage and GC content, organized in columns (Fig. 2A) with the option to sort by a selected column. These records are uniquely identified by a locus identifier (ORF ID) encompassing ‘N’ (for *Neisseria*) followed by a six-digit number representing individual CDSs and RNAs. These identifiers of all the CDSs and RNAs are hyperlinked and users can click to access the corresponding information page in NCBI GenBank Database enabling them to access the original manually curated annotations for their reference. To facilitate a better visualization of the results, each page by default displays 100 records. Users can access them by searching on their order around the genome or even by the functional classification of their gene product (Figs. 2B and 2C). NeisseriaBase is also equipped with a number of analytical tools such as sequence similarity search tools, in-house designed tools such as PathoProT for comparative pathogenomics analysis and PGC tool for comparing whole-genomes.

### Pathogenomic analysis of the *Neisseria* strains using the PathoProT tool

Virulence factors or genes, broadly classified into two major categories of pathogenesis (toxigenesis and invasiveness), enable the pathogenic organism to establish itself on or within a host thereby increasing its chances of causing disease (Chen et al., 2005; Yang et al., 2008; Chen et al., 2012). As such, virulence factors are important targets for novel drugs and design of new vaccines (Peterson, 1996). This inspired us to develop the comprehensive PathoProT capable of distinctively predicting and comparing the associative virulence genes for user-defined strains of *Neisseria* species according to the threshold customized by the users.

**Figure 3:**
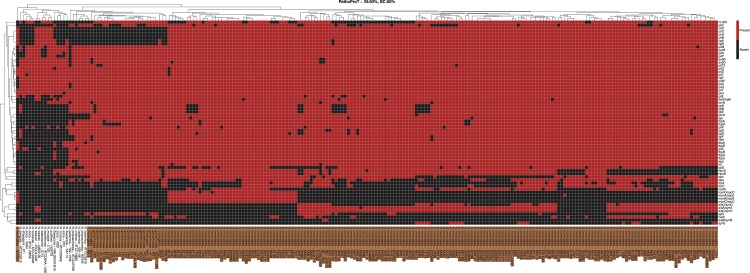
The heatmap showing the cluster of *Neisseria* strains based on virulence genes, generated using PathoProT with the threshold of 50% sequence identity and 50% sequence completeness. The columns represent the *Neisseria* strains, whereas the rows represent the predicted virulence genes. A red box indicates the presence of the virulence gene in the corresponding strain while a black box indicates absence of the gene. Cluster trees built based on the virulence factors composition are shown on each side of the map margins. The pathogenic strains are highlighted in brown.

To demonstrate the utility of the PathoProT, the pipeline was first tested using the threshold of 50% sequence identity and sequence completeness to predict possible virulence genes and cluster the *Neisseria* strains in NeisseriaBase based on their virulence profiles as shown in a heatmap generated using the PathoProT of the *Neisseria* genus in Fig. 3. Interestingly, we found that the virulence genes shared by almost all the strains of the pathogenic species *N. meningitidis and N. gonorrhoeae* were genes encoding the pili proteins, a type of polymeric pericellular glycoproteins which exhibit hair-like projections and function to establish initial attachment in order to promote adhesion to host tissues (Virji, 2009). It was observed that *pilE, pilS, pilC* and *pilX* were present in both *N. meningitidis* and *N. gonorrhoeae*, as well as few commensal species. The other *pil* genes were present in almost all the *Neisseria* species (even in the commensal species). The analysis also revealed that a group of virulence genes *ctrA, ctrB, ctrC, ctrD*, *lipA* and *lipB*, were found to be present in most *N. meningitidis* strains and few commensal species, but absent in most *N. gonorrhoeae* strains. The *ctrB* gene is responsible for encoding the hydrophobic inner membrane proteins, whereas *ctrC* accounts for the integral inner membrane-associated protein in the ABC (ATP binding cassette) transport system. *lipA* and *lipB*, also referred to as *ctrE* and *ctrF* respectively, play a major role in stimulating surface expression of an anchored capsule polymer (Roberts, 1996). These polysaccharide capsules are major virulence factors for *N. meningitidis* and antigenic differences in the polysaccharide capsules are the key in *in-vitro* serogrouping of these bacteria (Murray, Rosenthal & Pfaller, 2012). *N. gonorrhoeae*, on the other hand does not possess a true carbohydrate capsule but has a capsule-like negative charge (Murray, Rosenthal & Pfaller, 2012).

**Figure 4:**
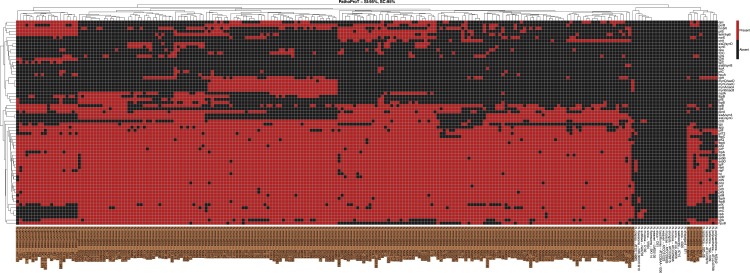
The heatmap showing the cluster of *Neisseria* strains based on virulence genes, generated using PathoProT with stringent threshold of 95% sequence identity and 95% sequence completeness. The columns represent the *Neisseria* strains, whereas the rows represent the predicted virulence genes. A red box indicates the presence of the virulence gene in the corresponding strain while a black box indicates absence of the gene. Cluster trees built based on the virulence factors composition are shown on each side of the map margins. The pathogenic strains are highlighted in brown.

We also tested the PathoProT pipeline using highly stringent cutoff of 95% sequence identity and sequence completeness. The generated heatmap clearly differentiated the pathogenic species of *Neisseria* from the non-pathogenic species, which did not show the presence of any of the known virulence genes (Fig. 4). As can be observed from the Fig. 4, the two major subunits (*pilE* and *pilS*) and minor subunit, *pilC* are harboured by only the known pathogenic species which are *N. meningitidis* and *N. gonorrhoeae*. Moreover, several minor subunits of pili proteins *(pilD, pilF, pilG, pilM, pilN, pilO, pilP pilQ, pilT, pilU, pilX and pilW*) were present only in the pathogenic clades and only two other commensal species, *N. lactamica* and *N. polysaccharea*. In fact, these two commensals are the closest species to the two known pathogenic species by DNA relatedness (Guibourdenche, Popoff & Riou, 1986; Marri et al., 2010; Bennett, Jolley & Maiden, 2013). The *pilE* gene which encodes the main subunit that makes up the pilus fibre, has undergone intergenomic and intragenomic variations where recombinase A-dependent recombination events occurred between one of numerous *pilS*, silent pilin genes and pile, expressed pilin gene. This antigenic variation of pilin is believed to give rise to the significant survival niche of *Neisseria* pathogenic species, presumably developing better adaptation to the changing external environment (Heckels, 1989). Furthermore, it is usually unlikely for commensal species to perform antigenic shift of *pilE/pilS* recombination due to the deficiency of guanine-repeat element at the region upstream of *pilE*. Even with a rigorous threshold of 95% sequence identity and sequence completeness, we still observed the group of virulence genes (*ctrB, ctrC, lipA* and *lipB*) were present in most of the *N. meningitidis* strains but were absent from the other *Neisseria* species including the pathogenic species *N. gonorrhoeae*. Moreover, the analysis also revealed that one of the genes specific for pathogenic *Neisseria* genomes which is rarely found in commensals is the *iga*, which is only present in *N. meningitidis* and *N. gonorrhoeae*. This *iga* gene encodes an IgA protease which plays a role in cleaving secretory IgA in the hinge region (Marri et al., 2010).

### Genome comparison and visualisation using the PGC tool

For the efficient comparative genomic analysis between the *Neisseria* genomes either cross-strain or cross-species, we have developed an automated pipeline named PGC. Through this analytical tool, users can choose two genomes of interest from the NeisseriaBase, or users can upload their own genome sequence and compare with one of the strains in NeisseriaBase through the custom submission form for the whole-genome comparison, which will then be processed by the PGC tool. One of the genomes will be the reference genome while the other one the query genome for comparison purposes.

**Figure 5:**
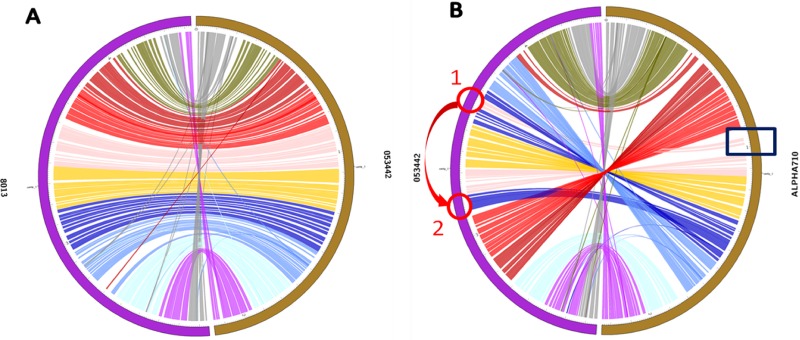
Pairwise genome comparison. (A) Circos-generated PGC plot of genome comparison between strains 053442 (right) and 8013 (left); (B) Circos generated PGC plot of genome comparison between strains alpha710 (right) and 053442 (left). The red arrow indicates the translocation that occurred from position 1 to position 2. The black box indicates the location of prophages.

To illustrate the functionality of our PGC pipeline, we present two examples of pairwise alignment. The complete genome sequence of *N. meningitidis* 053442 was aligned with two other strains of *N. meningitidis*: 8013 and alpha710. The parameters for both the pairwise alignment comparisons were set to 95% of minimum identity and a LT of 5,000 base pairs. The PGC plot between *N. meningitidis* 053442 and *N. meningitidis* 8013 is shown in Fig. 5A while the PGC plot between *N. meningitidis* 053442 and *N. meningitidis* alpha710 is shown in Fig. 5B.

**Figure 6:**
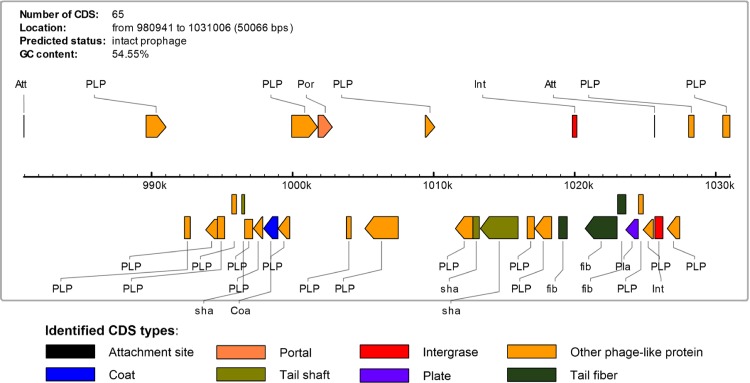
Linear prophage view of the intact prophage of alpha710 strain.

As evident from Fig. 5A, no significant crosslink was found across the PGC plot between *N. meningitidis* strains 053442 and 8013, instead a wide range of ribbon spread was detected. On the other hand, we can observe that *N. meningitidis* strains 053442 and alpha710 share most of the similar genomic elements indicated by the ribbons with respect to their position, size, and orientation in Fig. 5B. However, there is one remarkable genome rearrangement found in the PGC plot shown by the blue ribbon labeled in Fig. 5B. This genome element of *N. meningitidis* alpha710, is believed to have been translocated from position 1 to position 2 in *N. meningitidis* 053442 as indicated by red circles in the Fig. 5B. We also observed that there was significant genome dissimilarities (gaps) found in the *N. meningitidis* alpha710, the region marked with black box in the Fig. 5B. In order to determine its source of origin, we utilized the web server tool named PHAST (PHAge Search Tool) (Zhou et al., 2011) in order to identify, annotate and provide possible prophage sequences and visualization within both the *Neisseria* genomes. Based on the PHAST search, three prophages were identified in the aplha710 genome; an intact prophage (score: 140), an incomplete prophage (score: 40) and a questionable prophage (score: 80). All three prophages were closely arranged with each other and all three of them were present in that particular genome region of *N. meningitidis* alpha710 marked with the black box in Fig. 5B. The predicted complete prophage consisted of 65 CDS, having a size of 50,066 base pairs. The GC content of the complete prophage was 54.55%. The phage proteins encoded within this region were Int, phage-encoded *λ* integrase protein; Por, portal protein; fib, tail fiber and Pla, plate (Fig. 6). Based on these observations from the PGC plot and the PHAST results, we propose that the *N. meningitidis* alpha710 genome strain might have undergone insertion with prophages leading to the particular genome dissimilarities with *N. meningitidis* 053442 genome.

### Visualising *Neisseria* genome data

A Javascript-based genome browser known as JBrowse (Skinner & Holmes, 2010) has been incorporated into NeisseriaBase to facilitate the display of *Neisseria* genomes and annotations by supporting fast and smooth animated genome navigation over the web. This latest lightweight AJAX-based genome browser enables the user to navigate the selected genome providing smooth and efficient panning and zooming of a genomic region in the genome of a *Neisseria* species via embedded navigation buttons. The built-in JBrowse enables visualization of the CDSs and RNAs within a particular contig along with the relevant information such as its type, function or subsystem classification and its start and stop positions (Fig. 2C).

Furthermore, the database search menu enables the users to perform a direct search for strain information by applying query filters either by strain type, relevant keywords within the selected species and strain or simply by the identifiers. Additionally, all the strain records such as genome assembly, annotations, CDS or RNA sequences are available for download from the online resources.

## Discussion

The two prominent species of the *Neisseria* genus, *N. gonorrhoeae* causative agent of gonorrhoea (Kenneth, 2012) and *N. meningitidis* responsible for meningococcal infection, are major pathogens in humans (Morse, 1996; Chen et al., 2005; Yang et al., 2008; Chen et al., 2012). Over the years, these two species and their different isolates have been a well-researched topic and yet, there is still a lot to be discovered. This has resulted in the development of a number of databases dedicated to these two species of *Neisseria* providing valuable information and insights to the *Neisseria* research community. There is a database which provides research and clinical aspects of the two above mentioned pathogenic species of *Neisseria* genus (http://neisseria.org/); *Neisseria* database resources which involve Multilocus Sequence Typing (MLST) study of *Neisseria* alleles and genes (www.pubmlst.org/neisseria) (Jolley & Maiden, 2010), *Neisseria* associated disease, and meningococcal pathological research. A specialised database specifically for *N*. *meningitidis* genomes is maintained, to characterize the genomes of an increasing number of virulent and non-virulent *N. meningitidis* and elucidate the genetic causes of virulence of this species of *Neisseria* (Katz et al., 2011). The genome study of *N. meningitidis* serogroup A strain Z2491, *N. meningitidis* serogroup C strain FAM18 and *N. lactamica* 020-06 are presented in the Scientific Resources of the Sanger Institute which is funded by the Wellcome Trust/Beowulf Genomics (www.sanger.ac.uk/resources/downloads/bacteria/neisseria.html). Another biological resource is NeMeSys (Rusniok et al., 2009), providing useful tools and mutagenesis studies for a serogroup C clinical isolate (strain 8013) (Geoffroy et al., 2003). There is an existing database, which is specific to *N. gonorrhoeae* (http://stdgen.northwestern.edu/stdgen/bacteria/ngon/). Broad Institute of Havard and MIT maintains resources specific to *N. gonorrhoeae* under their *Neisseria gonorrhoeae* group Sequencing Project along with some of the other *Neisseria* species (www.broadinstitute.org). Apart from this, the Comprehensive Antibiotic Resistance Database (CARD) has incorporated *Neisseria* associated antibiotics and their targets along with antibiotic resistance genes, proteins, and related literature. PATRIC (Wattam et al., 2014) is another database, providing genomic and virulence factors information of some of the *Neisseria* strains, however it does not provide functionalities for comparing, clustering and visualizing the virulence gene profiles for comparative pathogenomic analysis.

A full understanding of the *Neisseria* genus requires tools that also include the commensal species. Extensive research of this genus has shown that *Neisseria* has the capability of acquisition and incorporation of genetic material specifically through horizontal gene transfer (Knapp, 1988; Saez Nieto, Marcos & Vindel, 1998; Qvarnstrom & Swedberg, 2006; Maiden, 2008; Higashi et al., 2011; Rotman & Seifert, 2014) which has contributed to its rich diversity and genetic variation. It has been proposed that sometimes the acquisition of individual virulence factors by non-pathogenic bacteria may convert them into a pathogen (Hensel et al., 1995). Research has shown that *N. elongata* and *N. flavescens* considered to be non-pathogenic forms of *Neisseria* (in humans) have the potential of being opportunistic human pathogens, causing severe and sometimes fatal disease in humans (Coovadia, 1984; Hofstad, Hope & Falsen, 1998; Hombrouck-Alet et al., 2003). This clearly indicates that the other species of *Neisseria* should no more be neglected or ignored as a possible cause of disease.

With the advances in high-throughput sequencing technologies, as more *Neisseria* genomes are sequenced, we need to study, characterize and more importantly compare them to understand their biology and their evolution. In order to do that, we can no longer just concentrate on the two known pathogenic species, but it is necessary also to consider the *Neisseria* genus as a whole. With that idea in mind we have developed the freely available specialised *Neisseria* genus biological database NeisseriaBase. This database is dedicated to *Neisseria* research and provides genomic information for all the *Neisseria* species (both pathogenic species and commensal species) along with a host of features and in-house designed tools to facilitate the easy access and high quality comparative analysis of these genome data. At the moment, NeisseriaBase comprises of 227 *Neisseria* genome sequences with 603,500 CDSs, 16,071 RNAs and 13,119 tRNA genes.

The database is organised and presented in such a way that the user can conveniently access assimilate and explore the desired data or information in an intuitive manner. The database is equipped with features like the JBrowse allowing a smooth genome browsing and real-time search option for direct searching for strain information by applying query filters. NeisseriaBase provides the option to download all the strain records, using the download option provided in the “Download” page. Additionally, analytical tools such as similarity search tools like BLAST and VFDB BLAST and in-house developed PGC and PathoProT incorporated in the NeisseriaBase will allow the researchers to perform high quality comparative analysis providing first hand insight into the genomes of the *Neisseria* strains.

Genomic information from a single *Neisseria* genome may not always be enough to give us a clear understanding of its biology and extended view of the gene pool of the species. A comparative genomic study of multiple *Neisseria* genomes will help us to determine the relatedness and the variations between the organisms at the genetic level e.g., indels and rearrangements between two genomes either inter- or intra-species. It will also help to identify the evolutionary signals and potential regions associated with pathogenicity, thus enabling us to study the evolutionary changes among organisms, identify the orthologous genes in species, as well as genes that give each organism its unique characteristics. This prompted us to develop and incorporate the PGC tool in the NeisseriaBase. Although PGC is inspired from similar tools, like Circoletto (Darzentas, 2010) and RCircos (Zhang, Meltzer & Davis, 2013), it has distinct advantages over its predecessors. PGC uses NUCmer (global alignment) package in MUMmer, as its alignment algorithm for it is more apt for large-scale and rapid genome alignment, whereas in Circoletto alignments are based on BLAST (local alignment). PGC provides an online form which allows the users to adjust the minimum percent genome identity (%), merging of links/ribbons according to MT, and the removal of links according to the user-defined LT. A histogram track showing the percentage of mapped regions along the genomes is added in the circular layout generated by PGC, helping the users to identify putative indels and repetitive regions in the compared genomes. RCircos, developed using R packages, has similar functionality but requires a base knowledge in R programming for running the package. By contrast, PGC provides a user-friendly interface and easy to use add-on marks, requiring no prior knowledge in programming languages for running the package giving an edge over RCircos.

Identification of potential virulence factors is very important as it serves as an indicator for the pathogenicity of bacteria as well as a critical contributor for identification of targets for novel drugs and design of new vaccines (Peterson, 1996). PathoProT was developed by in-house Perl and R scripts and incorporated into NeisseriaBase specifically for the purpose of identifying the potential genes that express virulence factors in the *Neisseria* genomes. The PathoProT analysis will result in the hierarchical clustering of the identified virulence genes in the selected *Neisseria* stains and the data can be visualized in the form of a heat map of multiple virulence gene profiles.

### Conclusions

NeisseriaBase is aimed to providing a user-friendly database that not only serves access to a host of genomic resources of *Neisseria*, but will also enable the user to perform high-quality comparative genome analysis. NeisseriaBase will be maintained and updated regularly to provide the most accurate and detailed information about *Neisseria* genus. This database will provide an important platform for the expanding scientific community interested in *Neisseria* research. To accelerate the development of this NeisseriaBase for the use of the scientific community, suggestions on improving this database and requests for additional functions are most welcome.

### Future directions

We will be checking for the availability of new *Neisseria* genomes on a consistent basis in order to update and insert the new available genomes into the NeisseriaBase. Additionally, latest publications and upcoming events regarding *Neisseria* will be regularly updated in the homepage of the database. We will be working on enhancing the functionality of the existing bioinformatics tools in the NeisseriaBase for providing a better analytical platform. Other researchers or research groups are welcome to email us at girg@um.edu.my if they opt to share their annotations, opinions, and curated data with us.

## Supplemental Information

10.7717/peerj.1698/supp-1 Figure S1 Pathogenomics Profiling Tool (PathoProT) workflow

## List of Abbreviations

CDSs: Coding Sequences
ORFs: Open Reading Frames
NCBI: National Center for Biotechnology Information
VFDB: Virulence Factor Database
MVC: Model View Controller
AJAX: Asynchronous JavaScript and XML
PGC: Pairwise Genome Comparison
PathoProT: Pathogenomics Profiling Tool
RAST: Rapid Annotation using Subsystem Technology
MLST: Multilocus Sequence Typing
PHAST: PHAge Search Tool

## Additional Information and Declarations

### Data Availability

The following information was supplied regarding data availability: NeisseriaBase: http://neisseria.um.edu.my.
