# Supplementary material for: NeisseriaBase: a specialised Neisseria genomic resource and analysis platform
Source: PeerJ. 2016 Mar 17;4:e1698. doi: 10.7717/peerj.1698 (PMC4806638; doi:10.7717/peerj.1698)
Supplement: Figure S1 [file peerj-04-1698-s001.pdf]

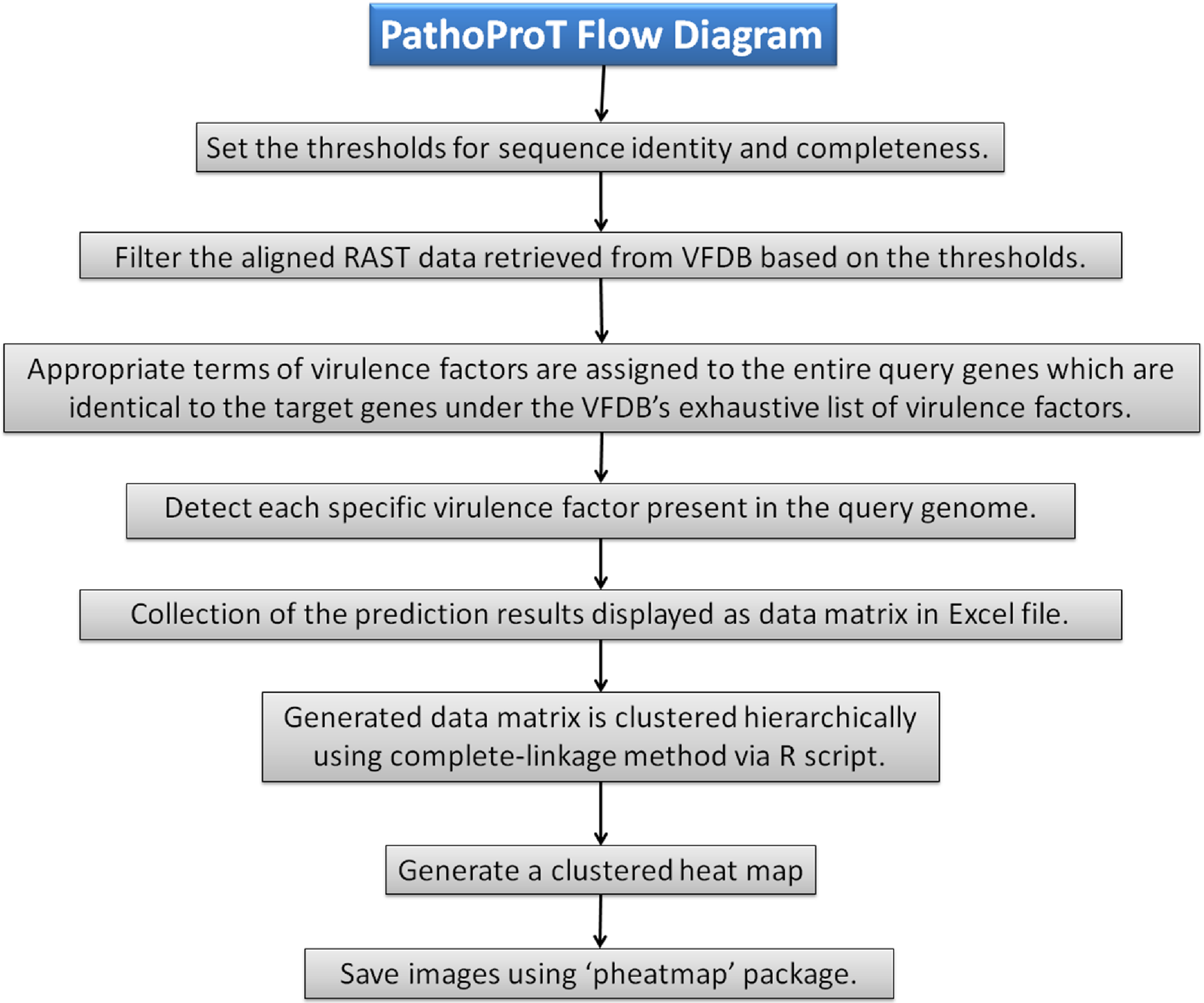
PathoProT Flow Diagram
Set the thresholds for sequence identity and completeness.
Filter the aligned RAST data retrieved from VFDB based on the thresholds.
Appropriate terms of virulence factors are assigned to the entire query genes which are identical to the target genes under the VFDB's exhaustive list of virulence factors.
Detect each specific virulence factor present in the query genome.
Collection of the prediction results displayed as data matrix in Excel file.
Generated data matrix is clustered hierarchically using complete-linkage method via R script.
Generate a clustered heat map
Save images using 'pheatmap' package.
